# Quantification of phytoplankton bloom dynamics by citizen scientists in urban and peri-urban environments

**DOI:** 10.1007/s10661-015-4912-9

**Published:** 2015-10-15

**Authors:** Eva Pintado Castilla, Davi Gasparini Fernandes Cunha, Fred Wang Fat Lee, Steven Loiselle, Kin Chung Ho, Charlotte Hall

**Affiliations:** Earthwatch Institute, 256 Banbury Road, Oxford, UK; Departamento de Hidráulica e Saneamento, Escola de Engenharia de São Carlos, Universidade de São Paulo, São Carlos, SP Brazil; School of Science and Technology, The Open University of Hong Kong, Hong Kong, China

**Keywords:** Phytoplankton, Algal bloom monitoring, Urban ecosystems, Citizen science

## Abstract

Freshwater ecosystems are severely threatened by urban development and agricultural intensification. Increased occurrence of algal blooms is a main issue, and the identification of local dynamics and drivers is hampered by a lack of field data. In this study, data from 13 cities (250 water bodies) were used to examine the capacity of trained community members to assess elevated phytoplankton densities in urban and peri-urban freshwater ecosystems. Coincident nutrient concentrations and land use observations were used to examine possible drivers of algal blooms. Measurements made by participants showed a good relationship to standard laboratory measurements of phytoplankton density, in particular in pond and lake ecosystems. Links between high phytoplankton density and nutrients (mainly phosphate) were observed. Microscale observations of pollution sources and catchment scale estimates of land cover both influenced the occurrence of algal blooms. The acquisition of environmental data by committed and trained community members represents a major opportunity to support agency monitoring programmes and to complement field campaigns in the study of catchment dynamics.

**Electronic supplementary material:**

The online version of this article (doi:10.1007/s10661-015-4912-9) contains supplementary material, which is available to authorized users.

## Introduction

Freshwater systems are highly threatened by a range of anthropogenic activities including intensive agriculture, urbanisation, industrialisation and land cover change (Meybeck 2003). These pressures affect all ecosystem services provided by these systems, including water supply for human consumption, food production and industry, fishing, flood protection and recreational activities (Vorosmarty et al. 2005; Waltham et al. 2014). Vorosmarty et al. (2010) found that almost 80 % of the world’s population live in areas with high risk for human water security and biodiversity.

The growing human population combined with the increasing tendency to live in cities has led to an increase in the number of streams flowing through urbanised areas (Meyer et al. 2005). The effects of urbanisation on water bodies have been described as “urban stream syndrome” and include alterations in geomorphology and hydrology, decrease in biodiversity, dominance of toxic, tolerant and invasive species and increase in the concentrations of organic compounds, nutrients and algal biomass (Meyer et al. 2005; Walsh et al. 2005; Millington et al. 2015; Halstead et al. 2014). Artificial eutrophication constitutes one of the main threats to aquatic ecosystems worldwide, especially in urban catchments (Smith et al. 1999; Taylor et al. 2004; Carpenter 2005).

Excessive nutrient loads from point and non-point sources cause shifts in the frequency and duration of phytoplankton growth and development, harmful algal blooms and the formation of hypoxic and/or anoxic conditions (Conley et al. 2009; MacLeod et al. 2011; Huang et al. 2014). Anthropogenic activities, such as fertiliser and detergent use, land use change, industrial sewerage and leaking septic systems play a key role in increasing these loads (Sylvan et al. 2007; Howarth 2008; Nyenje et al. 2010). Which nutrient plays the limiting role to algal blooms, usually nitrogen (N) or phosphorus (P), is a controversial subject (Conley et al. 2009; Huszar et al. 2006), and seasonal and spatial variations occur (Malone et al. 1996). Most commonly, P is considered to be the main limiting nutrient (Schindler et al. 2008; Conley et al. 2009), but N limitation (Ryther and Dunstan 1971; Elser et al. 1990; James et al. 2003) or NP co-limitation (Cunha and Calijuri 2011; Lee et al. 2015) also occurs. In addition, the effect of light availability also has to be considered (Cunha et al. 2012).

Studies to identify the temporal and spatial dynamics of algal blooms and the impacts of urbanisation on water bodies have been carried out at different spatial scales (e.g. Booth et al. 2004; Hatt et al. 2004; Roy et al. 2005; Lee 2000). Large-scale studies are hindered by the lack of field data to determine the frequency and duration of the algal blooms as well as the identification of local micro-scale conditions of land use.

Citizen science, also known as civic or community science (Kruger and Shannon 2000; Carr 2004), refers to the involvement of citizens on research, mainly with data collection (e.g. Canfield et al. 2002; Nicholson et al. 2002; Turner and Richter 2011; Donnelly et al. 2014), although level of engagement and tasks vary and might include result interpretation and analyses (e.g. Cardamone et al. 2009; Conrad and Hilchey 2011; Khatib et al. 2011; Macknick and Enders 2012; Lee et al. 2014). Citizen science projects have increased in importance and scope over the last decade (Miller-Rushing et al. 2012; Sauermann and Franzoni 2015) as a cost-effective alternative for acquiring high-resolution data and information. The most known case is the role of volunteers in the ornithological field which can be traced back to the eighteenth century (Greenwood 2007), but there are also same examples in aquatic science (e.g. Lowry and Fienen 2013; Buytaert et al. 2014; Lottig et al. 2014).

In this study, data from 250 urban and peri-urban water bodies were used to examine the capacity of citizen scientists to assess high phytoplankton densities. Quantitative measurements and qualitative observations were analysed together with laboratory-based measurements of phytoplankton density to (1) determine if elevated phytoplankton density can be detected by citizens and (2) study possible drivers of eutrophication and algal blooms in urban catchments. To our knowledge, this study represents the first analysis of the community-based monitoring of algal bloom dynamics and their drivers.

## Methods

This study has been carried out at two different scales: global, using data from 13 cities of the FreshWater Watch (FWW) database, and local, with more detailed measurements from three cities (São Paulo and Curitiba in Brazil and Hong Kong in China), where simultaneous samples of phytoplankton for laboratory analysis were also obtained.

### Data acquisition

The present study used data obtained from trained citizen scientists who actively participated in the FWW. The FWW database includes more than measurements from 1,650 streams, rivers, lakes and ponds from 25 urban and peri-urban areas across the globe. Data were obtained following a consistent methodology and were quality controlled using side by side measurements, laboratory measurements and scientist/non-scientist comparisons. Data were uploaded directly online by participants, quality checked and in some cases corrected by the same citizen scientists after notification of inconsistent data inputs. All participants went through identical training sessions in which sampling, measurements, data acquisition, data upload and analysis were addressed using classroom and field exercises. Following training, multi-language online support was provided to maintain understanding, feedback and engagement.

Thirteen cities were chosen to be included in the study, from which only sites with at least three measurements were considered in the analysis. The cities were selected for a balanced geographical distribution: Boston, Buffalo and Chicago (USA), Buenos Aires (Argentina), Curitiba, São Paulo and Rio de Janeiro (Brazil), Delhi (India), Guangzhou and Hong Kong (China), Mexico D.F. (Mexico), Jakarta (Indonesia) and Vancouver (Canada). There were a total of 2,048 measurements (1,390 lotic and 658 lentic). Data were collected between April 2013 and September 2014 by teams of trained citizen scientists. For every sample, 16 qualitative and quantitative variables were recorded (see supplementary information). In the present evaluation, algae presence, turbidity, water colour, the presence of pollution sources as well as the concentrations of phosphate and nitrate were analysed.

Algae presence was recorded as a qualitative variable; volunteers chose from one of the following options by a drop-down menu with photographic support: no algae, evenly dispersed algae, floating mats, attached algae or blue-green scum.

Turbidity was determined using calibrated Secchi tubes (14–240 NTU) (Tyler 1968; Preisendorfer 1986; Wernand 2010). Secchi depth has been successfully used in citizen science programmes (Lathrop et al. 1996; Bruhn and Soranno 2005; Lottig et al. 2014) and has provided a high accuracy when compared to measurements taken by professional scientists (Obrecht et al. 1998; Canfield et al. 2002).

Water colour measurements were obtained as categorical data, recorded as the colour perceived by the citizen scientists using a drop-down menu which included the following selections: colourless, yellow, brown, green or other (specifying which colour). Water colour has been used as an index to assess water quality for more than a century (Mortimer 1958) to estimate changes in dissolved organic matter (Cuthbert and Del Giorgio 1992). The use of upwelling radiance to estimate algal biomass and the concentrations of photosynthetic pigments is the basis for ocean colour remote sensing (e.g. Gitelson et al. 1993; Olmanson et al. 2013; Duan et al. 2014a). Visual measurements using a colour scale (eg. Forel-Ule scale) have been used in citizen science-related studies (Novoa et al. 2014).

To determine the major drivers of eutrophication and algal blooms, the relationships between phytoplankton and nutrients (N-NO_3_, P-PO_4_), land use and pollution sources were analysed.

Nitrate concentrations were estimated colourimetrically using *N*-(1-napthyl)-ethylenediamine (Adeloju 2013) in seven specific ranges from 0.2 to 10 mg/L N-NO_3_ (Kyoritsu Chemical, Tokyo, Japan). Phosphate concentrations were estimated colourimetrically using inosine enzymatic reactions in seven specific ranges from 0.02 to 1.0 mg/L P-PO_4_ (Strickland and Parsons 1968).

Pollution sources were recorded in number and type using a drop-down menu, choosing from industrial discharge, residential discharge, urban/road discharge and other (specifying type and location).

All datasets included geographical coordinates and sampling time obtained using the FWW smartphone application or online tools. Selection of water body type (stream, river, pond, lake or other) was also made using common local water body name.

For comparative purposes, professionally obtained measurements of phytoplankton density were also undertaken in rivers and streams (all lotic systems) in São Paulo, Curitiba and Hong Kong. In Brazil, water samples (*n* = 56) were collected in July and October 2013 and February 2014, preserved with Lugol’s iodine solution and analysed in the Laboratory BIOTACE at the University of São Paulo. The organisms were counted through sedimentation chambers (Utermöhl 1958) in an inverted microscope (Olympus CK2®), with their densities expressed as organisms per milliliter (Eaton et al. 2005). In Hong Kong, water samples (*n* = 132) were collected between May 2014 and December 2014 and also fixed with Lugol’s solution. The sample was concentrated by natural sedimentation, and density was determined by directly counting under a light microscope using a Sedgwick-Rafter counting cell.

Global land cover data from the Food and Agriculture Organization (FAO) Global Land Cover SHARE (GLC-SHARE) database (Latham et al. 2014) and global watershed boundaries from HydroBASINS (Lehner and Grill 2013) were used to calculate the percentage of surface covered by each land cover class for every sampling site’s watershed. The FAO GLC-SHARE includes land cover classes of artificial surfaces, cropland, grassland, tree-covered area, shrub-covered area, herbaceous vegetation, mangroves, sparse vegetation, bare soil, water bodies and snow and glaciers. The HydroBASINS’s watershed boundaries were developed on behalf of the World Wildlife Fund (Lehner and Grill 2013) and have already been used in several freshwater ecological studies (Carrizo et al. 2013; Markovic et al. 2014; Grill et al. 2015).

### Data analysis

Water quality data did not meet the requirements for parametric statistics; therefore, all tests used in this study are non-parametric and were made with IBM SPSS Statistics 21.

All measurements were divided in two groups, according to the presence or absence of algae. It was considered that algae were present when algal characteristics of evenly dispersed, floating mats or blue-green scum were recorded and absent when no algae or attached algae were recorded (since most sampled water bodies presented aquatic vegetation on the bottom). These two groups were compared in terms of water colour, turbidity, phosphate and nitrate concentrations, presence of pollution sources and land use through Mann–Whitney tests. Additionally, Spearman’s rank was calculated between algae presence and all variables. This analysis was carried out for all samples together and separately for rivers/streams and ponds/lakes.

For water colour, a number was assigned to each class to perform the analysis: 0 colourless, 1 yellow, 2 brown, 3 green and 4 other (since most “other” records referred to purple, grey or black). The number and type of local pollution sources were recorded on site and assigned a specific number (0 none, 1 urban/road discharge, 2 residential discharge and 3 industrial discharge). When more than one type of source was present, their correspondent values were summed. For the land cover analysis, the FAO land cover classes were combined in three main classes: artificial surface, crop land and vegetation (composed by shrubs, trees, sparse vegetation, herbaceous vegetation and grassland). The percentage of watershed surface covered by each of these classes was calculated using ArcGIS 10.2. Watershed delimitation was based on a Pfafstetter classification (Lehner and Grill 2013) and a level 8 was used (Markovic et al. 2014).

Phytoplankton samples from two Brazilian cities (São Paulo, Curitiba) and Hong Kong were divided in two groups, lower and higher phytoplankton density, according to the median values for each dataset as a reference, 14,593 org/mL and 420 org/mL, respectively. This division was not used to compare datasets from the study cities or to classify bloom and non-bloom conditions which require common data (phycocyanin or chlorophyll-*a* concentrations) that was not obtained. The dominant phytoplankton biomass was cyanobacteria in Curitiba and São Paolo, where study sites were higher order streams. Bacillariophyceae and Chlorophyceae dominated phytoplankton biomass in Hong Kong, where the study sites were lower order streams with lower residence time and shorter growing period.

## Results

### Observations of algal presence

**Fig. 1:**
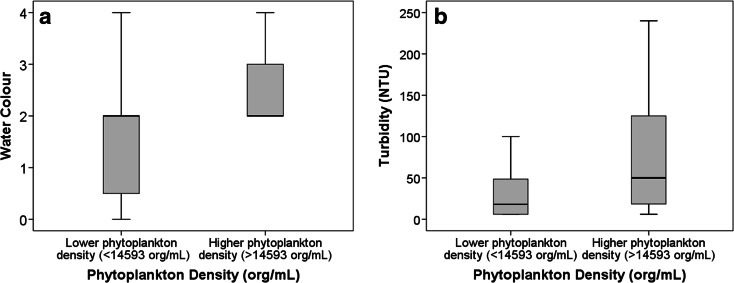
**a** Phytoplankton density (org/mL) versus water colour (water colour: *0* clear, *1* yellow, *2* brown, *3* green, *4* other) for samples from São Paulo and Curitiba (*n* = 56). **b** Phytoplankton density (org/mL) versus turbidity (NTU) for samples from São Paulo and Curitiba (*n* = 56)

**Fig. 2:**
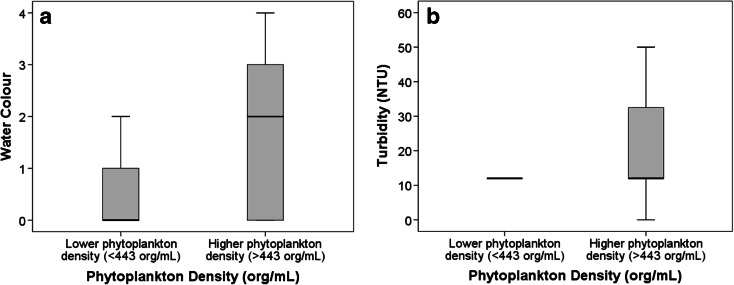
**a** Phytoplankton density (org/mL) versus water colour (water colour: *0* clear, *1* yellow, *2* brown, *3* green, *4* other) for samples from Hong Kong (*n* = 132). **b** Phytoplankton density (org/mL) versus turbidity (NTU) for samples from Hong Kong (*n* = 133)

Correlations with turbidity showed a strong relationship with phytoplankton density, 0.474 (*p* < 0.001) and 0.546 (*p* < 0.001) in São Paolo/Curitiba and Hong Kong, respectively. The low and high phytoplankton density categories had significantly different turbidity (Mann–Whitney, *p* = 0.012 for São Paulo/Curitiba and *p* < 0.001 for Hong Kong) (Figs. 1 and 2). Significant but lower relationships with water colour were observed (*p* = 0.023 for São Paulo and Curitiba and *p* < 0.001 for Hong Kong).

**Fig. 3:**
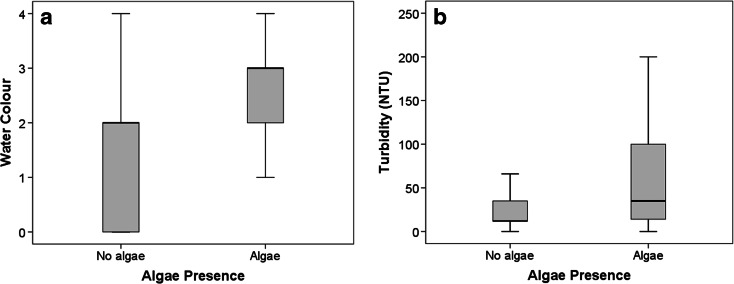
**a** Water colour versus algae presence for all sites (*n* = 2,048). Water colour: *0* clear, *1* yellow, *2* brown, *3* green, *4* other. **b** Turbidity versus algae presence for all sites (*n* = 2,048)

Citizen scientists’ observations of algae presence were found to correspond to a significant difference in water colour, with a higher possibility of positive algae presence being associated with a green water colour, compared to water bodies without observed algae presence (*p* < 0.001). Turbidity was higher in water bodies with observed algae presence (*p* < 0.001) (Fig. 3). Spearman’s rank was 0.245 for turbidity and 0.224 for water colour.

There was a higher correlation of algae presence with turbidity and water colour in lentic water bodies (Table 1) with respect to lotic water bodies. Turbidity showed stronger relationships with algal presence than water colour for both lotic and lentic sites.

**Table 1:** Summary of algae presence relationships with water colour and turbidity for all study ecosystems, lotic and lentic sites

	Water colour	Turbidity
All data	*p* < 0.001 *ρ* = 0.224 (*p* < 0.001)	*p* < 0.001 *ρ* = 0.245 (*p* < 0.001)
Lotic	*p* < 0.001 *ρ* = 0.115 (*p* < 0.001)	*p* < 0.001 *ρ* = 0.134 (*p* < 0.001)
Lentic	*p* < 0.001 *ρ* = 0.295 (*p* < 0.001)	*p* < 0.001 *ρ* = 0.364 (*p* < 0.001)

First row of each cell corresponds to the Mann–Whitney test’s *p* value and the second one to the Spearman’s rank (*ρ*)

### Relationship between phytoplankton and nutrients

**Fig. 4:**
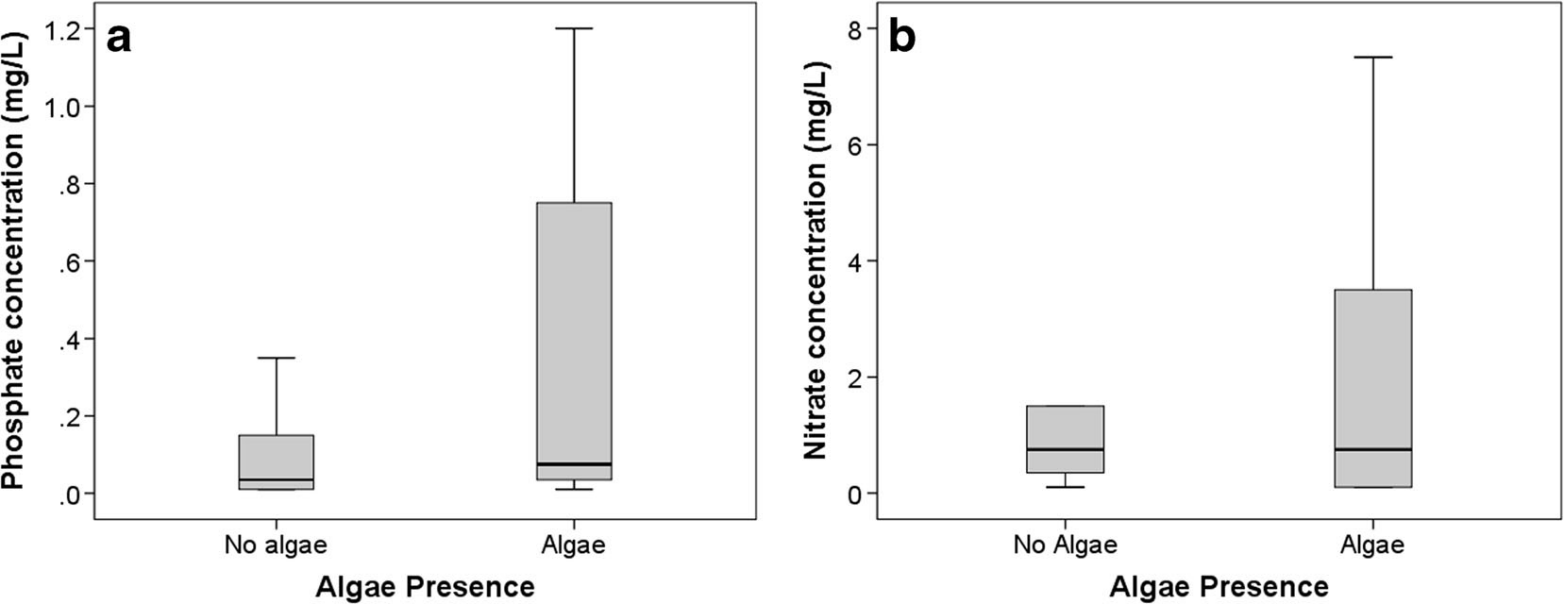
**a** Algae presence versus phosphate concentration (mg/L) for all sites (*n* = 2,048). **b** Algae presence versus nitrate concentration (mg/L) for all sites (*n* = 2,048)

The comparison between citizen scientist-acquired measurements of nutrients and their simultaneous observations of algae presence showed clear differences between N-NO_3_ and P-PO_4_. Phosphate concentrations were significantly higher when algae presence was observed (*p* < 0.001) for pooled data from lotic and lentic water bodies (Fig. 4). No significant relationship was found between algae presence and nitrate concentration (Mann–Whitney *p* = 0.096, Spearman’s *ρ* = 0.037). The analysis for lentic water bodies showed significant relationships between algae presence and both phosphate and nitrate concentrations (Table 2). Lotic water bodies showed a relationship between algae presence and phosphate concentration only (Table 2). This relationship was consistent with measurements of phytoplankton density and phosphate concentrations in São Paulo/Curitiba and Hong Kong, where significant relationships where found between phytoplankton density and phosphate (*p* < 0.001 and *p* = 0.001, respectively) with Spearman of 0.548 and 0.341. Correlations between phytoplankton density and nitrate concentrations were not significant (*p* > 0.05).

**Table 2:** Summary of relationships between algae presence and phosphate and nitrate concentrations

	Phosphate	Nitrate
All data	*p* < 0.001 *ρ* = 0.144 (*p* < 0.001)	*p* = 0.096 *ρ* = 0.037 (*p* = 0.096)
Lotic	*p* = 0.041 *ρ* = 0.055 (*p* = 0.041)	*p* = 0.424 *ρ* = 0.021 (*p* = 0.424)
Lentic	*p* < 0.001 *ρ* = 0.203 (*p* < 0.001)	*p* < 0.001 *ρ* = 0.162 (*p* < 0.001)

First row of each cell corresponds to the Mann–Whitney test’s *p* value and the second one to the Spearman’s rank (*ρ*)

### Relationships between phytoplankton, global and local land use data

**Fig. 5:**
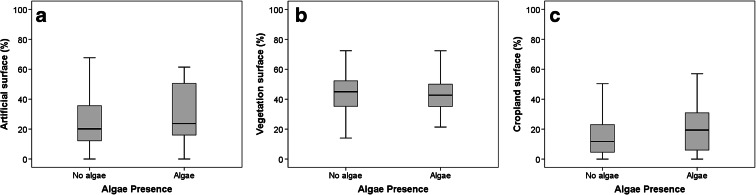
**a** Algae presence versus percentage of watershed surface covered by artificial structures for all sites. **b** Algae presence versus percentage of watershed surface covered by vegetation for all sites. **c** Algae presence versus percentage of watershed surface covered by cropland for all sites

Significant relationships were found between algae presence and all three land cover classes (Mann–Whitney, *p* < 0.001) (Fig. 5). Increased cropland and artificial surface and decreased vegetated surface characterised the sites where algal presence was highest. Artificial surface cover led to a significant difference in algae presence. When only lakes and ponds were considered, a significant relationship was found between algae presence and vegetated surfaces (Mann–Whitney *p* < 0.001, Spearman’s *ρ* = 0.237). Cropland coverage in lotic water bodies was significantly different for water bodies with and without algae presence (Table 3).

**Table 3:** Summary of watershed land cover and algae presence relationships, for all study ecosystems, lotic and lentic sites

	Artificial surface	Vegetated surface	Cropland
All data	*p* < 0.001 *ρ* = 0.096 (*p* < 0.001)	*p* < 0.001 *ρ* = 0.096 (*p* < 0.001)	*p* < 0.001 *ρ* = 0.101 (*p* < 0.001)
Lotic	*p* = 0.001 *ρ* = 0.087 (*p* = 0.001)	*p* = 0.928 *ρ* = 0.002 (*p* = 0.928)	*p* = 0.016 *ρ* = 0.065 (*p* = 0.016)
Lentic	*p* < 0.001 *ρ* = 0.188 (*p* < 0.001)	*p* < 0.001 *ρ* = 0.237 (*p* < 0.001)	*p* = 0.949 *ρ* = 0.003 (*p* = 0.945)

First row of each cell corresponds to the Mann–Whitney test’s *p* value and the second one to the Spearman’s rank (*ρ*)

**Fig. 6:**
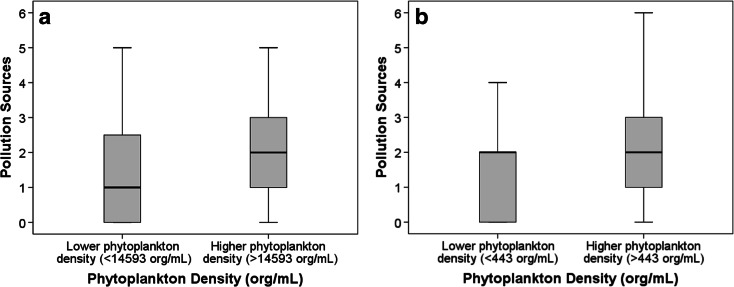
**a** Phytoplankton density (org/mL) versus pollution sources for São Paulo and Curitiba (*n* = 56). **b** Phytoplankton density (org/mL) versus pollution sources for Hong Kong (*n* = 132). Pollution sources: *0* none, *1* urban/road discharge, *2* residential discharge, *3* industrial discharge

The number of observed local pollution sources was related to significant differences between the algae and non-algae groups (*p* = 0.012) when all water body types were considered. Lentic sites showed a stronger correlation between the citizen-observed local sources (*p* = 0.009), although Spearman correlations were quite low (*ρ* = 0.055 and *ρ* = 0.070, respectively). When analysing the relationship between pollution sources and phytoplankton density in Brazil and Hong Kong, a significant difference was found between the groups with higher and lower phytoplankton densities (Fig. 6, *p* = 0.045 and *p* = 0.007, respectively) with a Spearman correlation of *ρ* = 0.297 (*p* = 0.028) and *ρ* = 0.261 (*p* = 0.003), respectively.

## Discussion

Observations of algae presence and laboratory measurements of phytoplankton density were well correlated to quantitative (turbidity) and qualitative (water colour) measurements, suggesting that trained community members can make qualitative estimates of increased phytoplankton density. Of the three indicators of algae presence, turbidity provided the best accuracy. This was verified in the global dataset of lotic and lentic water bodies, as well as the pooled dataset of both water body types. As a quantitative measurement, correlations for turbidity were higher for phytoplankton densities in São Paulo, Curitiba and Hong Kong than to algae presence in all sites.

By separating the global dataset into lentic and lotic water bodies, information on turbidity and colour showed different levels of significance. This is a natural consequence of the structural, hydrological and functional differences between these kinds of ecosystems. The lower correlation in lotic water bodies was most likely due to the additional presence of resuspended particulate matter in stream and river environments (Prestigiacomo et al. 2007). On the other hand, lower turbulence, increased sedimentation of inorganic particles and increased light availability favour the dominance of phytoplankton biomass in turbidity measurements and estimates of water colour in ponds and lakes (Duan et al. 2014b). Higher phytoplankton density in lentic ecosystems was evidenced by all three indicators of algae presence.

Another factor is the increased difficulty of estimating water colour in moving waters because of the more complex surface texture and reflectance, which is strongly influenced by local flow conditions (Carbonneau and Piégay 2012). It should be noted that imprecisions associated to visual observations are inevitable (e.g. Williams et al. 2006; Cooper et al. 2007), in particular when two qualitative variables that are visual manifestations of the same phenomenon are recorded (phytoplankton-rich waters being assigned to a green water colour).

For the pooled lentic and lotic data, significant relationships were found between phosphate concentrations and both algae presence and phytoplankton density, whilst no significant relationship was found with nitrate concentrations. This correlation with phosphate was higher for phytoplankton density data, which could be associated by the improved performance of Spearman’s rank for continuous variables. With respect to differences in water bodies, phosphate showed a stronger correlation with algae presence in lentic with respect to lotic systems. Additionally, lentic systems presented a significant but lower correlation between algae presence and nitrate concentration. In lotic water bodies, elevated vertical mixing, lower water residence time and higher ratios of bankside vegetation to open water area create conditions where light limitation may control phytoplankton densities, in particular in the smaller streams which dominated the present study. The relative importance of nutrient or light limitation is expected to vary seasonally and spatially with respect to changes in phytoplankton community, nutrient loads and condition of stratification as well as light conditions (Conley et al. 2009; Loiselle et al. 2008; Yue et al. 2014).

The presence of algae was affected differently by local pollution sources and land use. Lentic sites presented a significant relationship with local pollution sources (low correlation), whilst no relationship was found for lotic ecosystems. Ponds and small lakes are expected to be more sensitive to local sources of pollution as residence time is higher and mixing is lower. The results from the streams examined in São Paulo and Curitiba and Hong Kong showed a positive relationship between local pollution sources and phytoplankton density, in particular in the Brazilian streams, where residence time was lower.

The effects of land cover on algae presence occurrence suggested that sites with greater percentages of cropland and artificial surface favoured higher phytoplankton density. For lentic ecosystems, correlations between algae presence and vegetated and artificial surfaces were relatively high (0.237 and 0.188, respectively). Lotic ecosystems showed a significant positive relationship with cropland and artificial surface percentages, although correlations were low (0.065 and 0.087). These relationships were limited by the low resolution of the land use information used, suggesting the need of complementary high-resolution (local) land use data.

## Conclusions

Trained citizen scientists made effective observations of algae presence across a wide range of environments and ecosystems. This information could improve detection of changes in phytoplankton dynamics in urban/peri-urban water bodies as well as provide complementary data for statuary agency monitoring (e.g. early warning). Of the information acquired, turbidity was found to provide the best indication of elevated phytoplankton densities with respect to observations of water colour. The accuracy of citizen acquired data was best for lentic systems where biogenic turbidity probably dominated.

Citizen-acquired information on pollution sources also provided useful information for predicting algal blooms. Likewise, low-resolution land use information showed links between local catchment conditions and the occurrence of high phytoplankton biomass. Combining both levels of information might be appropriate for water management and artificial eutrophication control.

Microalgae observations and measurements followed expected differences between lotic and lentic ecosystems, in relation to light availability, biogeochemistry and hydrology. Lentic ecosystems had the highest frequency of algae presence and were the most sensitive to nutrient concentrations. In particular, phosphate concentrations covaried with phytoplankton biomass in both lentic and lotic environments.

In the present study, more than 2,000 datasets were obtained by citizen scientists, an equivalent of thousands of hours of effort that scientists were not required to make to obtain this information. These data can be used as complementary information to field campaigns in the development water quality models or their validation. The identification of harmful species and possible toxin production could provide an early warning to statutory agencies in urban/peri-urban areas. The growing interest and willingness of committed citizen scientists to undertake these activities represent a major opportunity to improve our understanding and management of these important ecosystems.

## Electronic supplementary material

ESM 1 (PDF 83 kb)

## References

[CR1] Adeloju SB (2013). Progress and recent advances in phosphate sensors: a review. Talanta.

[CR2] Booth DB, Karr JR, Schauman S, Konrad CP, Morley SA, Larson MG (2004). Reviving urban streams: land use, hydrology, biology, and human behaviour. Journal of the American Water Resources Association.

[CR3] Bruhn L, Soranno P (2005). Long term (1974–2001) volunteer monitoring of water clarity trends in Michigan lakes and their relation to ecoregion and land use/cover. Lake and Reservoir Management.

[CR4] Buytaert W, Zulkafli Z, Grainger S, Acosta L, Alemie TC, Bastiaensen J (2014). Citizen science in hydrology and water resources: opportunities for knowledge generation, ecosystem service management, and sustainable development. Hydrosphere.

[CR5] Canfield DE, Brown CD, Bachmann RW, Hoyer MV (2002). Volunteer lake monitoring: testing the reliability of data collected by the Florida LAKEWATCH program. Lake and Reservoir Management.

[CR6] Carbonneau P, Piégay H (2012). Fluvial remote sensing for science and management.

[CR7] Cardamone C, Schawinski K, Sarzi M, Bamford SP, Bennert N, Urry CM (2009). Galaxy Zoo Green Peas: discovery of a class of compact extremely star-forming galaxies. Monthly Notices of the Royal Astronomical Society.

[CR8] Carpenter SR (2005). Eutrophication of aquatic ecosystems: bistability and soil phosphorus. Proceedings of the National Academy of Sciences of the United States of America.

[CR9] Carr AJL (2004). Policy reviews and essays. Society & Natural Resources.

[CR10] Carrizo SF, Smith KG, Darwall WRT (2013). Progress towards a global assessment of the status of freshwater fishes (Pisces) for the IUCN Red List: application to conservation programmes in zoos and aquariums. International Zoo Yearbook.

[CR11] Conley DJ, Paerl HW, Howarth RW, Boesch DF, Seitzinger SP, Havens KE (2009). Ecology. Controlling eutrophication: nitrogen and phosphorus. Science (New York, N.Y.).

[CR12] Conrad CC, Hilchey KG (2011). A review of citizen science and community-based environmental monitoring: issues and opportunities. Environmental Monitoring and Assessment.

[CR13] Cooper C, Dickinson J, Phillips T, Bonney R (2007). Ecology and society: citizen science as a tool for conservation in residential ecosystems. Ecology and Society.

[CR14] Cunha DGF, Calijuri MDC (2011). Limiting factors for phytoplankton growth in subtropical reservoirs: the effect of light and nutrient availability in different longitudinal compartments. Lake and Reservoir Management.

[CR15] Cunha DGF, Bottino F, Calijuri MDC (2012). Can free-floating and emerged macrophytes influence the density and diversity of phytoplankton in subtropical reservoirs?. Lake and Reservoir Management.

[CR16] Cuthbert ID, Del Giorgio P (1992). Toward a standard method of measuring color in freshwater. Limnology and Oceanography.

[CR17] Donnelly A, Crowe O, Regan E, Begley S, Caffarra A (2014). The role of citizen science in monitoring biodiversity in Ireland. International Journal of Biometeorology.

[CR18] Duan H, Feng L, Ma R, Zhang Y, Loiselle SA (2014). Variability of particulate organic carbon in inland waters observed from MODIS Aqua imagery. Environmental Research Letters.

[CR19] Duan H., Ma R., Loiselle S.A., Shen Q., Yin H. & Zhang Y. (2014b). Optical characterization of black water blooms in eutrophic waters. *The Science of the total environment* 482–483, 174–83. 10.1016/j.scitotenv.2014.02.113 24657365

[CR20] Eaton, A. D., Clesceri, L. S., Rice, E. W., Greenberg, A. E., & Franson, M. A. H. (2005). APHA: standard methods for the examination of water and wastewater. Centennial Edition., APHA, AWWA, WEF, Washington, DC.

[CR21] Elser JJ, Marzolf ER, Goldman CR (1990). Phosphorus and nitrogen limitation of phytoplankton growth in the freshwaters of North America: a review and critique of experimental enrichments. Canadian Journal of Fisheries and Aquatic Sciences.

[CR22] Gitelson A, Garbuzov G, Szilagyi F, Mittenzwey KH, Karnieli A, Kaiser A (1993). Quantitative remote sensing methods for real-time monitoring of inland waters quality. International Journal of Remote Sensing.

[CR23] Greenwood JJD (2007). Citizens, science and bird conservation. Journal of Ornithology.

[CR24] Grill G, Lehner B, Lumsdon AE, Macdonald GK, Zar C (2015). An index-based framework for assessing patterns and trends in river fragmentation and flow regulation by global dams at multiple scales. Environmental Research Letters.

[CR25] Halstead JA, Kliman S, Berheide CW, Chaucer A, Cock-Esteb A (2014). Urban stream syndrome in a small, lightly developed watershed: a statistical analysis of water chemistry parameters, land use patterns, and natural sources. Environmental Monitoring and Assessment.

[CR26] Hatt BE, Fletcher TD, Walsh CJ, Taylor SL (2004). The influence of urban density and drainage infrastructure on the concentrations and loads of pollutants in small streams. Environmental Management.

[CR27] Howarth RW (2008). Coastal nitrogen pollution: a review of sources and trends globally and regionally. Harmful Algae.

[CR28] Huang C., Wang X., Yang H., Li Y., Wang Y., Chen X., et al. (2014). Satellite data regarding the eutrophication response to human activities in the plateau lake Dianchi in China from 1974 to 2009. *Science of the Total Environment* 485–486, 1–11. 10.1016/j.scitotenv.2014.03.031 24698830

[CR29] Huszar VLM, Caraco NF, Roland F, Cole J (2006). Nutrient–chlorophyll relationships in tropical–subtropical lakes: do temperate models fit?. Biogeochemistry.

[CR30] James C, Fisher J, Moss B (2003). Nitrogen driven lakes: the Shropshire and Cheshire Meres?. Archiv für Hydrobiologie.

[CR31] Khatib F, DiMaio F, Cooper S, Kazmierczyk M, Gilski M, Krzywda S (2011). Crystal structure of a monomeric retroviral protease solved by protein folding game players. Nature Structural & Molecular Biology.

[CR32] Kruger LE, Shannon MA (2000). Getting to know ourselves and our places through participation in civic social assessment. Society & Natural Resources.

[CR33] Latham J., Cumani R., Rosati I., & Bloise M. (2014). FAO Global Land Cover (GLC-SHARE) Beta-Release 1.0 Database, Land and Water Division. Data is available at www.glcn.org/index_en.jsp.

[CR34] Lathrop RC, Carpenter SR, Rudstam LG (1996). Water clarity in Lake Mendota since 1900: responses to differing levels of nutrients and herbivory. Canadian Journal of Fisheries and Aquatic Sciences.

[CR35] Lee J (2000). Characterization of urban stormwater runoff. Water Research.

[CR36] Lee J, Kladwang W, Lee M, Cantu D, Azizyan M, Kim H (2014). RNA design rules from a massive open laboratory. Proceedings of the National Academy of Sciences of the United States of America.

[CR37] Lee TA, Rollwagen-Bollens G, Bollens SM (2015). The influence of water quality variables on cyanobacterial blooms and phytoplankton community composition in a shallow temperate lake. Environmental Monitoring and Assessment.

[CR38] Lehner B. & Grill G. (2013). Global river hydrography and network routing: baseline data and new approaches to study the world’s large river systems. *Hydrological Processes* 27, 2171–2186. Data is available at www.hydrosheds.org.

[CR39] Loiselle SA, Azza N, Cozar A, Bracchini L, Tognazzi A, Dattilo A (2008). Variability in factors causing light attenuation in Lake Victoria. Freshwater Biology.

[CR40] Lottig NR, Wagner T, Norton HE, Spence CK, Webster KE, Downing JA (2014). Long-term citizen-collected data reveal geographical patterns and temporal trends in lake water clarity. PLoS ONE.

[CR41] Lowry CS, Fienen MN (2013). Crowd hydrology: crowdsourcing hydrologic data and engaging citizen scientists. Ground Water.

[CR42] Macknick JE, Enders SK (2012). Transboundary forestry and water management in Nicaragua and Honduras: from conflicts to opportunities for cooperation. Journal of Sustainable Forestry.

[CR43] MacLeod A, Sibert R, Snyder C, Koretsky CM (2011). Eutrophication and salinization of urban and rural kettle lakes in Kalamazoo and Barry Counties, Michigan, USA. Applied Geochemistry.

[CR44] Malone TC, Conley DJ, Fisher TR, Glibert PM, Harding LW, Sellner KG (1996). Scales of nutrient-limited phytoplankton productivity in Chesapeake Bay. Estuaries.

[CR45] Markovic D, Carrizo S, Freyhof J, Cid N, Lengyel S, Scholz M (2014). Europe’s freshwater biodiversity under climate change: distribution shifts and conservation needs. Diversity and Distributions.

[CR46] Meybeck M (2003). Global analysis of river systems: from Earth system controls to Anthropocene syndromes. Philosophical Transactions of the Royal Society of London. Series B, Biological Sciences.

[CR47] Meyer JL, Paul MJ, Taulbee WK (2005). Stream ecosystem function in urbanizing landscapes published by : the North American Benthological Society Stream ecosystem function in urbanizing landscapes. Society.

[CR48] Miller-Rushing A, Primack R, Bonney R (2012). The history of public participation in ecological research. Frontiers in Ecology and the Environment.

[CR49] Millington HK, Lovell JE, Lovell CAK (2015). A framework for guiding the management of urban stream health. Ecological Economics.

[CR50] Mortimer, C. (1958) A treatise on limnology, vol. 1. Geography, physics, and chemistry. Chapman & Hall, London.

[CR51] Nicholson E, Ryan J, Hodgkins D (2002). Community data—where does the value lie? Assessing confidence limits of community collected water quality data. Water Science and Technology.

[CR52] Novoa S, Wernand MR, van der Woerd HJ (2014). The modern Forel-Ule scale: a ‘do-it-yourself’ colour comparator for water monitoring. Journal of the European Optical Society: Rapid Publications.

[CR53] Nyenje PM, Foppen JW, Uhlenbrook S, Kulabako R, Muwanga A (2010). Eutrophication and nutrient release in urban areas of sub-Saharan Africa—a review. Science of the Total Environment.

[CR54] Obrecht DV, Milanick M, Perkins BD, Ready D, Jones JR (1998). Evaluation of data generated from lake samples collected by volunteers. Lake and Reservoir Management.

[CR55] Olmanson LG, Brezonik PL, Bauer ME (2013). Airborne hyperspectral remote sensing to assess spatial distribution of water quality characteristics in large rivers: the Mississippi River and its tributaries in Minnesota. Remote Sensing of Environment.

[CR56] Preisendorfer RW (1986). Secchi disk science: visual optics of natural waters. Limnology and Oceanography.

[CR57] Prestigiacomo AR, Effler SW, O’Donnell D, Hassett JM, Michalenko EM, Lee Z (2007). Turbidity and suspended solids levels and loads in a sediment enriched stream: implications for impacted lotic and lentic ecosystems. Lake and Reservoir Management.

[CR58] Roy AH, Faust CL, Freeman MC, Meyer JL (2005). Reach-scale effects of riparian forest cover on urban stream ecosystems. Canadian Journal of Fisheries and Aquatic Sciences.

[CR59] Ryther JH, Dunstan WM (1971). Nitrogen, phosphorus, and eutrophication in the coastal marine environment. Science.

[CR60] Sauermann H, Franzoni C (2015). Crowd science user contribution patterns and their implications. Proceedings of the National Academy of Sciences.

[CR61] Schindler DW, Hecky RE, Findlay DL, Stainton MP, Parker BR, Paterson MJ (2008). Eutrophication of lakes cannot be controlled by reducing nitrogen input: results of a 37-year whole-ecosystem experiment. Proceedings of the National Academy of Sciences of the United States of America.

[CR62] Smith VH, Tilman GD, Nekola JC (1999). Eutrophication: impacts of excess nutrient inputs on freshwater, marine, and terrestrial ecosystems. Environmental Pollution.

[CR63] Strickland JDH, Parsons TR (1968). A practical handbook of seawater analysis.

[CR64] Sylvan JB, Quigg A, Tozzi S, Ammerman JW (2007). Eutrophication-induced phosphorus limitation in the Mississippi River plume: evidence from fast repetition rate fluorometry. Limnology and Oceanography.

[CR65] Taylor SL, Roberts SC, Walsh CJ, Hatt BE (2004). Catchment urbanisation and increased benthic algal biomass in streams: linking mechanisms to management. Freshwater Biology.

[CR66] Turner DS, Richter HE (2011). Wet/dry mapping: using citizen scientists to monitor the extent of perennial surface flow in dryland regions. Environmental Management.

[CR67] Tyler JE (1968). The Secchi disc. Limnology and Oceanography.

[CR68] Utermöhl H (1958). Zur Vervollkommnung der quantitativen Phytoplankton-Methodik. Limnology.

[CR69] Vorosmarty C.J., Bos R. & Balvanera P. (2005). Fresh water. In: *Ecosystems and human well-being: current state and trends,* pp. 165–207*.* UNEP Millennium Ecosystem Assessments Vol 1.

[CR70] Vorosmarty CJ, McIntyre PB, Gessner MO, Dudgeon D, Prusevich A, Green P (2010). Rivers in crisis: global water insecurity for humans and biodiversity. Nature.

[CR71] Walsh CJ, Roy AH, Feminella JW, Cottingham PD, Groffman PM, Morgan RP (2005). The urban stream syndrome: current knowledge and the search for a cure. Journal of the North American Benthological Society.

[CR72] Waltham, N. J., Reichelt-Brushett, A., McCann, D., & Eyre, B. D. (2014). Water and sediment quality, nutrient biochemistry and pollution loads in an urban freshwater lake: balancing human and ecological services. *Environmental Science: Processes & Impacts, 16*, 2804–2813. 10.1039/c4em00243a 25384753

[CR73] Wernand MR (2010). On the history of the Secchi disc. Journal of the European Optical Society: Rapid Publications.

[CR74] Williams ID, Walsh WJ, Tissot BN, Hallacher LE (2006). Impact of observers’ experience level on counts of fishes in underwater visual surveys. Marine Ecology Progress Series.

[CR75] Yue D, Peng Y, Qian X, Xiao L (2014). Spatial and seasonal patterns of size-fractionated phytoplankton growth in Lake Taihu. Journal of Plankton Research.

