# Supplementary material for: Quantification of phytoplankton bloom dynamics by citizen scientists in urban and peri-urban environments
Source: Environ Monit Assess. 2015 Oct 15;187(11):690. doi: 10.1007/s10661-015-4912-9 (PMC4607726; doi:10.1007/s10661-015-4912-9)
Supplement: Supplementary file 1 — (PDF 83 kb) [file 10661_2015_4912_MOESM1_ESM.pdf]

<u>Information recorded by citizen scientists in each dataset</u>

- Sample location (latitude/longitude)
- Site name
- Date/Time
- Observer name
- Type of freshwater body: pond/stream/lake/river/wetland/other
- Photograph of sampling site
- Surrounding land use (photographic supported drop down menu)
- Type of pollution sources (drop down menu)
- Evidence of the water uses (drop down menu)
- Identification of bank vegetation (drop down menu)
- Presence of aquatic life (drop down menu)
- Water (drop down menu)
- Water colour (drop down menu)
- Evidence of surface pollution
- Presence of elevated phytoplankton density or attached algae
- Concentrations of nitrate and phosphate
- Measurement of turbidity
